# Extremely strong infiltration of WT1-specific CTLs into mouse tumor by the combination vaccine with WT1-specific CTL and helper peptides

**DOI:** 10.18632/oncotarget.26338

**Published:** 2018-11-13

**Authors:** Jun Nakata, Hiroko Nakajima, Hiromu Hayashibara, Kanako Imafuku, Soyoko Morimoto, Fumihiro Fujiki, Daisuke Motooka, Daisuke Okuzaki, Kana Hasegawa, Naoki Hosen, Akihiro Tsuboi, Yoshihiro Oka, Atsushi Kumanogoh, Yusuke Oji, Haruo Sugiyama

**Affiliations:** ^1^ Department of Biomedical Informatics, Osaka University Graduate School of Medicine, Suita City, Osaka 565-0871, Japan; ^2^ Department of Cancer Immunology, Osaka University Graduate School of Medicine, Suita City, Osaka 565-0871, Japan; ^3^ Department of Cancer Immunotherapy, Osaka University Graduate School of Medicine, Suita City, Osaka 565-0871, Japan; ^4^ Genome Information Research Center, Research Institute for Microbial Diseases, Osaka University Graduate School of Medicine, Suita City, Osaka 565-0871, Japan; ^5^ Department of Cancer Stem Cell Biology, Osaka University Graduate School of Medicine, Suita City, Osaka 565-0871, Japan; ^6^ Department of Respiratory Medicine and Clinical Immunology, Osaka University Graduate School of Medicine, Suita City, Osaka 565-0871, Japan; ^7^ Department of Immunopathology, WP1 Immunology Frontier Research Center, Osaka University, Suita City, Osaka 565-0871, Japan; ^8^ Department of Functional Diagnostic Science, Osaka University Graduate School of Medicine, Suita City, Osaka 565-0871, Japan

**Keywords:** wilms tumor 1 (WT1), helper vaccine, cancer vaccine, resident memory T cells (T_RM_), tumor infiltrating lymphocytes (TIL)

## Abstract

In immunotherapy by cancer antigen-derived peptide vaccine, vaccination of cytotoxic T lymphocyte (CTL) peptide alone is common, while it remains unclear whether the addition of helper peptide vaccine to the CTL peptide vaccine is of great advantage for the enhancement of tumor immunity. In the present study, combination vaccine of Wilms’ tumor gene 1(WT1) protein-derived CTL and helper peptides induced the strong infiltration of WT1-specific CD8^+^ T cells into mouse tumor at frequencies of 8.8%, resulting in the formation of multiple microscopic necrotic lesions in the tumor, whereas the frequencies of WT1-specific CD8^+^ T cell infiltration into the tumor in the vaccination of the CTL peptide alone were only 0.32%. The majority of the infiltrated WT1-specific CD8^+^ T cells was effector phenotype T cells, but importantly, WT1-specific CD8^+^CD44^+^CD62L^+^CD103^+^ resident memory T cells, which could differentiate into a lot of effector phenotype T cells, existed in the tumor of mice vaccinated with the both WT1 peptides. Furthermore, T-cell receptor repertoire analysis showed the oligoclonality of these tumor infiltrating WT1 tetramer^+^ CD8^+^ T cells, and 3 clones occupied about half of them. These results indicated that WT1-specific CD4^+^ T cells played an essential role not only in the priming and activation of WT1-specific CD8^+^ T cells, but also in trafficking and infiltration of the CD8^+^ T cells into tumors. These results should provide us with the concept that in the clinical setting, combination vaccine of WT1-specific CTL and helper peptides would be more advantageous than the CTL peptide vaccine alone.

## INTRODUCTION

In the past two decades, immunotherapy became one of the established treatments against cancer as well as operation, radiotherapy and chemotherapy. However, so far, only a limited number of patients exhibit dramatic response, and even the PD-L1/PD-1 antibodies, which attracted the most attention in recent years, showed sufficient tumor shrinkage ranging only from 10 to 40% [1, 2]. The immunological analysis of tumors treated with the immunotherapies revealed the three basic immune profiles of tumors; the immune-inflamed, the immune-excluded, and the immune-desert phenotypes [3, 4]. And, from these observation of immune phenotypes, totally 7 steps of the cancer-immunity cycle, composed of cancer antigen release, cancer antigen presentation, priming and activation of T cells, trafficking of T cells to tumors, infiltration of T cells into tumor, recognition of cancer cells by T cells, and killing of cancer cells, were proposed to be necessary for the success in tumor immunotherapies [4]. Since every mono-therapies could target the specific one or a few steps, hundreds of clinical trials that combine several types of immunotherapies and other treatments are being attempted to exert these 7 steps effectively. Cancer tumor antigen-derived cytotoxic T lymphocyte (CTL) epitope-based vaccines target the specific steps of priming and activation of antigen-specific CD8^+^ CTLs, and combination with other therapies that target other steps is desired for the durable response. Helper peptide vaccines could be good candidates for combination with the CTL epitope-based vaccines, because the helper peptide vaccines are expected not only to help the step of priming and activation of cancer antigen-specific CTLs but also to stimulate the steps of trafficking and infiltration of the CTLs into tumors [5, 6].

The Wilms Tumor 1 (WT1) is a one of the most promising tumor associated antigens [7]. We and others have already performed many clinical studies using WT1 CTL peptide vaccine and reported the favorable clinical outcomes against many kinds of tumors [8–11]. WT1 is also the best target antigen for cancer immunotherapy in an *in vivo* experimental mouse model. Our group had already established a mouse WT1 immunotherapy model in which vaccination of mice with a WT1 CTL peptide (WT1_126–134_) could reject tumor transplanted in the mice [12]. As the extension of this study, we also reported that bacillus Calmette-Guérin cell wall skeleton (BCG-CWS) and interferon-β, which were used as adjuvants, could enhance the anti-tumor effect of WT1 CTL peptide vaccine [13, 14].

Recently, we identified a mouse WT1 protein-derived helper peptide (WT1_35–52_). Combination vaccine of WT1 CTL and the WT1 helper peptides could enhance and prolong the WT1-specific CTL response, compared to vaccination with the CTL peptide alone. Rejection rates of the transplanted tumors were 40% and 20% in mice treated with the combination vaccine and with the WT1 CTL peptide vaccination alone, respectively.

In the present study, we describe that combination vaccine of tumor-bearing mice with WT1-specific CTL and helper peptides induces very strong infiltration of WT1-specific CTLs and CD4^+^ T cells into the tumor, compared to the vaccination with WT1-specific CTL peptide alone, resulting in the formation of multiple microscopic necrotic lesions in the tumor. These results indicate that combination vaccine of tumor antigen-specific CTL and helper peptides is advantageous to promote strongly the immune response against tumor in immunotherapy.

## RESULTS

### Formation of microscopic necrotic lesions in the tumors of the mice co-vaccinated with WT1 CTL and helper peptides

**Figure 1:**
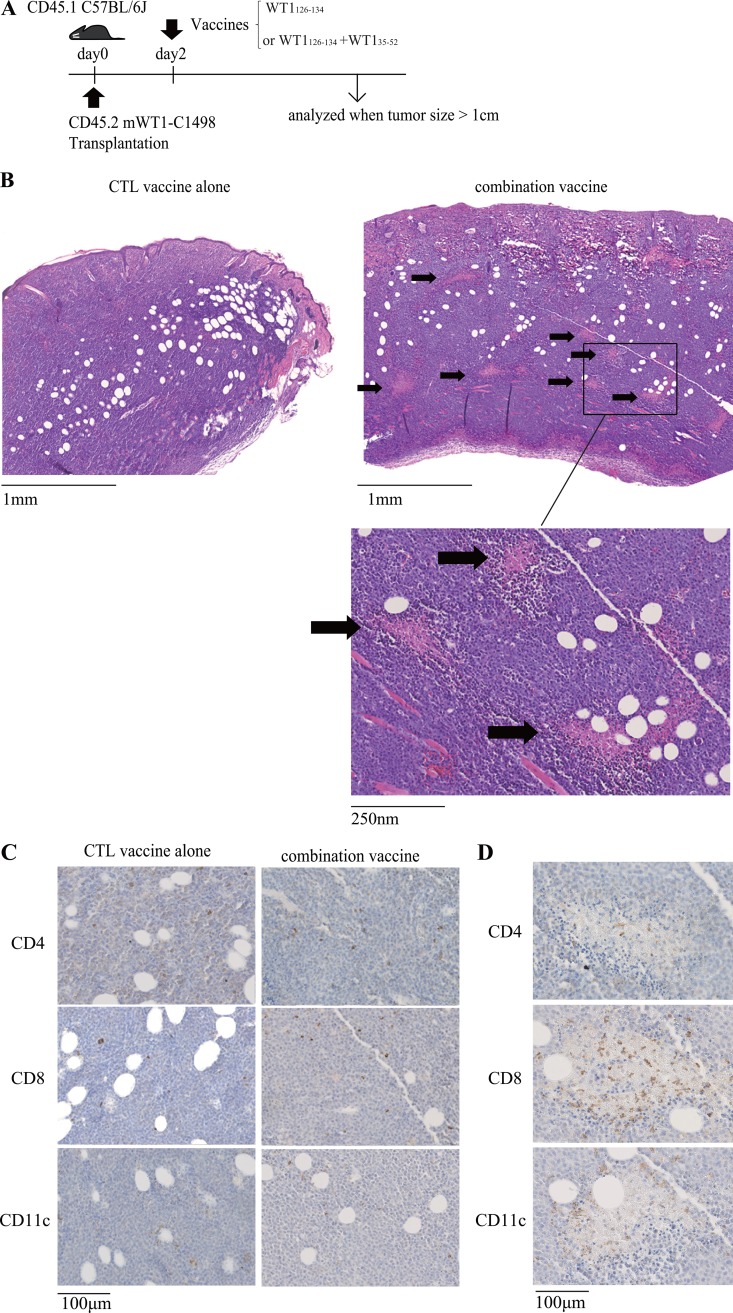
Formation of microscopic necrotic lesions in the tumors of the mice co-vaccinated with WT1 CTL and helper peptides (**A**) Tumor transplantation and vaccination schedule. WT1-expressing C1498 was subcutaneously transplanted on day 0, and WT1 CTL peptide alone or a mixture of WT1 CTL and helper peptides was administered on day 2. Tumors were analyzed when the tumor size reached over 1cm. (**B**) HE staining of subcutaneous tumors of the mice treated with the WT1 CTL peptide vaccine alone (left) or the combination vaccine with the WT1 CTL and helper peptides (right). The multiple microscopic necrotic lesions are shown by arrows. (**C**) Immuno-histochemical staining of tumors of the mice treated with the WT1 CTL peptide vaccine alone (left) or the combination vaccine with the WT1 CTL and helper peptides (right). (**D**) Representative results of the immuno-histochemical staining of a microscopic necrotic lesion in tumors of the mice treated with the combination vaccine.

Mice were subcutaneously transplanted with WT1-expressing C1498 leukemic cells on day 0 and vaccinated with WT1 CTL peptide alone or a mixture of WT1 CTL and helper peptides on day 2, and then tumors were resected for the pathological and immunological examination when they grew to a size of > 1 cm (Figure 1A). HE staining of the resected tumors revealed that substantial numbers of microscopic necrotic lesions (100 ∼ 300 μm) in the tumors were characteristically observed in the mice treated with the combination vaccine, but not detected in the mice vaccinated with WT1 CTL peptide alone (Figure 1B). Next, tumors were immuno-histochemically analyzed by CD4, CD8 and CD11c antibodies (Figure 1C). Although CD4^+^, CD8^+^ T cells and CD11c^+^ dendritic cells (DCs) similarly infiltrated into the tumors of both of the mice treated with the CTL peptide vaccine alone or the combination vaccine, the microscopic necrotic lesions had more CD8^+^ T cell infiltration (Figure 1D). Therefore, it appeared that the formation of the microscopic necrotic lesions resulted from CD8^+^ CTL-mediated immunological attack to tumors. Interestingly, CD11c^+^ DCs surrounded these microscopic necrotic lesions (Figure 1D). These results might raise the possibility that these CD11c^+^ DCs were involved in the infiltration of the CD8^+^ T cells into the microscopic necrotic lesions.

### Addition of the helper peptide on the CTL peptide-based vaccination increased the ratio of CD4^+^ T cells, but not the ratio of CD8^+^ T cells in tumors

**Figure 2:**
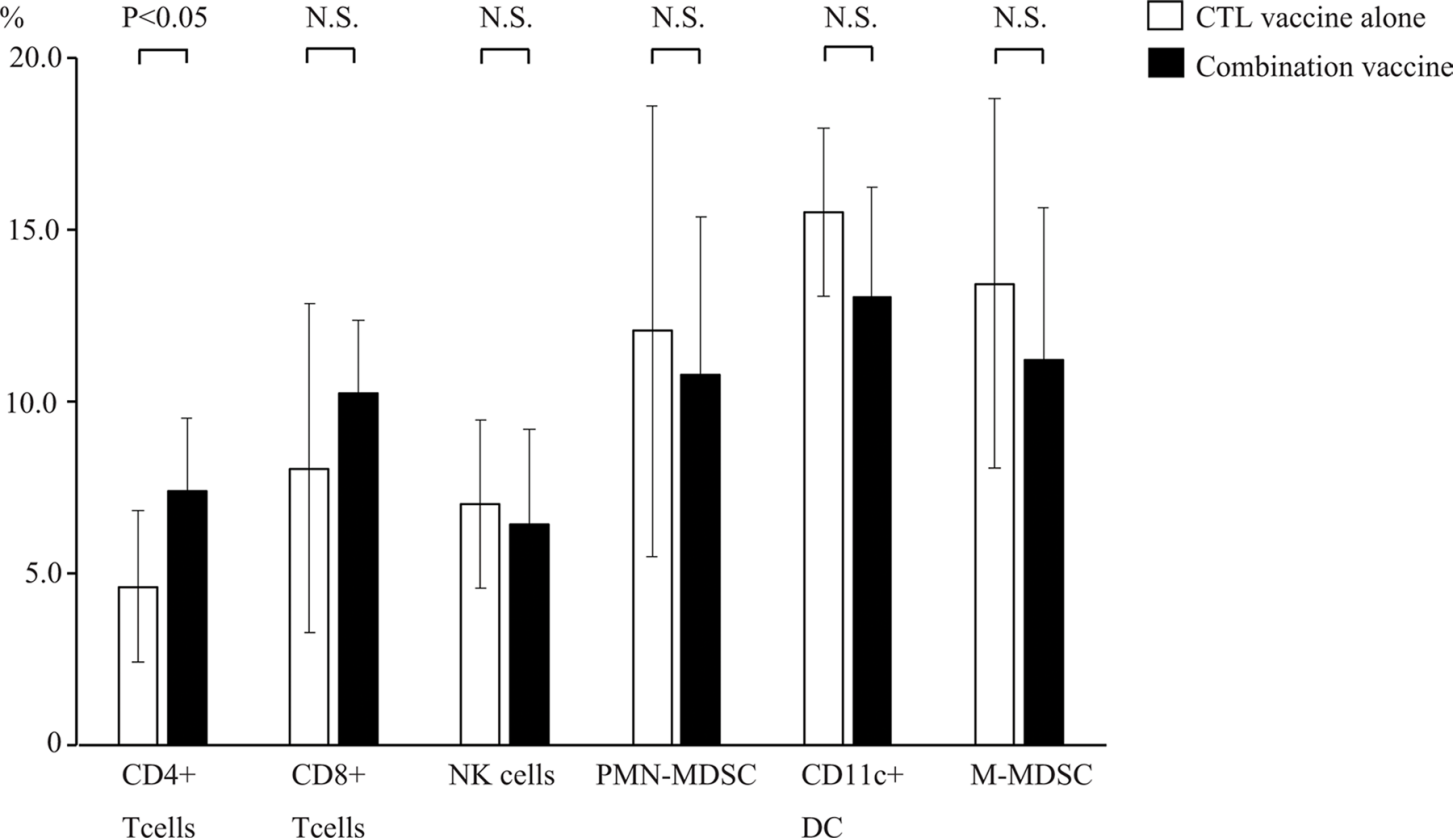
Frequencies of the tumor infiltrated immune cells Frequencies of CD4^+^ and CD8^+^ T cells, CD11b^+^Ly6G^+^ (PMN-MDSC), CD11c^+^ (DC) and CD11b^+^Ly6C^+^ (M-MDSC) cells among the tumor infiltration immune cells of mice treated with the WT1 CTL peptide vaccine alone (open bar, *n* = 8) or the combination vaccine with the WT1 CTL and helper peptides (solid bar, *n* = 8) are shown. Bar graphs show averages and standard deviations.

To examine the difference in immune response between the WT1 CTL peptide vaccine alone and the combination vaccine of the WT1 CTL and helper peptides, the tumor infiltrating immune cells were flow cytometrically analyzed. To distinguish immune cells from tumor cells, CD45.1/CD45.2 congenic mouse system was used. Tumor infiltrating CD45.1^+^ immune cells derived from recipient mice were positively selected by CD45.1^+^ Positive Selection Kit to discriminate from CD45.2^+^ tumor cells, and were then analyzed by flow cytometer for CD8^+^ and CD4^+^ T cells, CD11b^+^Ly6G^+^ (polymorphonuclear myeloid-derived suppressor cells (PMN-MDSC)), CD11c^+^ (dendritic cells (DC)) and CD11b^+^Ly6C^+^ (monocytic myeloid-derived suppressor cells (MDSC)) cells (Figure 2A). Only CD4^+^ T cells among the tumor infiltrating CD45.1^+^ immune cells were statistically higher in mice treated with the WT1 combination vaccine than in those treated with the WT1 CTL vaccine alone. On the contrary, CD8^+^ T cells among the tumor infiltrating CD45.1^+^ immune cells did not significantly increase in mice treated with the WT1 combination vaccine compared to those treated with the WT1 CTL vaccine alone. These results showed that CD4^+^ helper T cells more frequently infiltrated into the tumors of the mice treated with the WT1 combination vaccine, compared to those of the mice treated with the WT1 CTL peptide alone. It appeared that more frequent infiltration of the CD4^+^ T cells into the tumors of the mice treated with the WT1 combination vaccine resulted in the formation of microscopic necrotic lesions in the tumors.

### More frequent infiltration of WT1-specific CD8^+^ and CD4^+^ T cells into the tumors of mice treated with the WT1 combination vaccine

**Figure 3:**
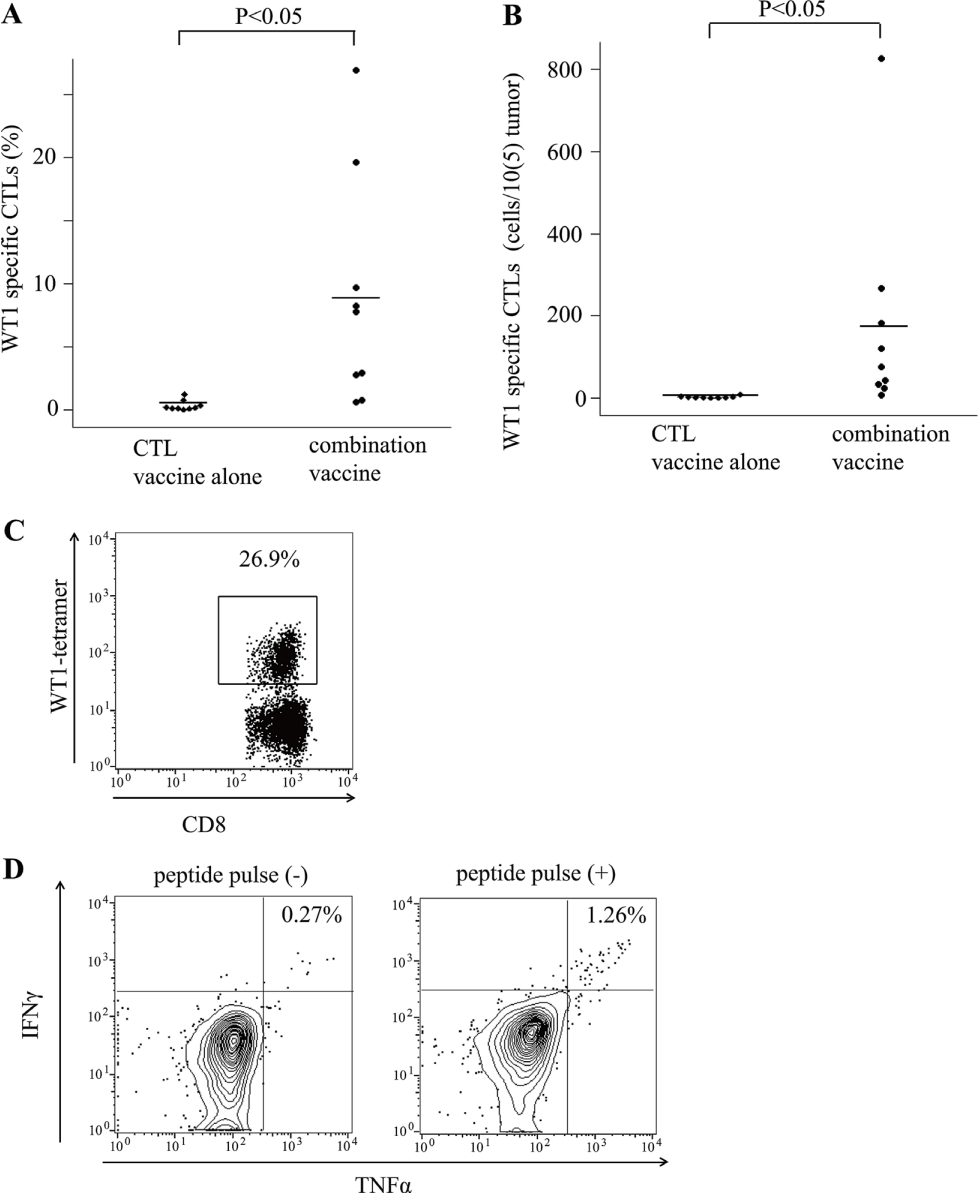
Tumor infiltrated WT1 tetramer^+^ CD8^+^ T cells (**A**) Bee swarm plots of the frequencies of WT1-tetramer^+^ CD8^+^ T cells among CD8^+^ T cells in tumors of the mice treated with the WT1 CTL peptide vaccine alone or the combination vaccine with the WT1 CTL and helper peptides. (**B**) Bee swarm plots of the number of WT1-tetramer^+^ CD8^+^ T cells per 1 × 10^5^ tumor cells. (**C**) A representative result of flow cytometry showing the high frequencies of WT1-tetramer^+^ CD8^+^ T cells among CD8^+^ T cells in tumors of the mice treated with the combination vaccine. (**D**) Representative results of flow cytometry of cytokine releasing assay of tumor infiltrating CD4^+^ T cells in mice treated with the combination vaccine. Splenocytes from a CD45.2 mouse pulsed or not pulsed with the WT1 helper peptide were used as the stimulator and control cells, respectively. The results of intracellular staining of IFNγ and TNFα of CD3^+^CD4^+^CD45.1^+^ T cells are shown.

Frequencies of WT1 tetramer^+^ CD8^+^ T cells were measured in tumor cell suspension that was made by disassembling the tumors. The frequencies of WT1 tetramer^+^ CD8^+^ T cells were much higher in the tumors of mice treated with the WT1 combination vaccine than in those of the mice treated with the WT1 CTL vaccine alone (8.8% versus 0.32%, *p* < 0.01, Figure 3A). The number of WT1 tetramer^+^ CD8^+^ T cells per 1 × 10^5^ tumor cells were also significantly higher in the tumors of mice treated with the WT1 combination vaccine than in those of mice treated with the WT1 CTL vaccine alone (Figure 3B). Representative dots of flow cytometry showed WT1 tetramer^+^ CD8^+^ T cells at frequencies of as high as 26.9% in tumors of mice treated with the WT1 combination vaccine (Figure 3C). Next, to examine the infiltration of WT1-specific helper CD4^+^ T cells into the tumors, tumor-infiltrated T cells (tumor cell suspension) were stimulated with WT1-specific helper peptide (WT135-52), which was used for the immunization of the mice, and WT1-specific helper peptide-specific TNF-α and/or IFN-γ production were analyzed by flow cytometry in CD3^+^ CD4^+^ T cells. Representative dot plots of TNF-α-and/or-IFN-γ-producing CD4^+^ T cells were shown in Figure 3D. These results indicated that addition of the WT1 helper peptide vaccine to the WT1 CTL peptide vaccine promoted the immune response against WT1-expressing tumors by the increased infiltration of WT1-specific CD4^+^ and CD8^+^ T cells into the tumor sites, especially by the strong infiltration of WT1-specific CD8^+^ T cells, resulting in the formation of microscopic necrotic lesions.

### Infiltration of WT1-specific resident memory T cells (T_RM_) into the tumors of mice treated with the WT1 combination vaccine

**Figure 4:**
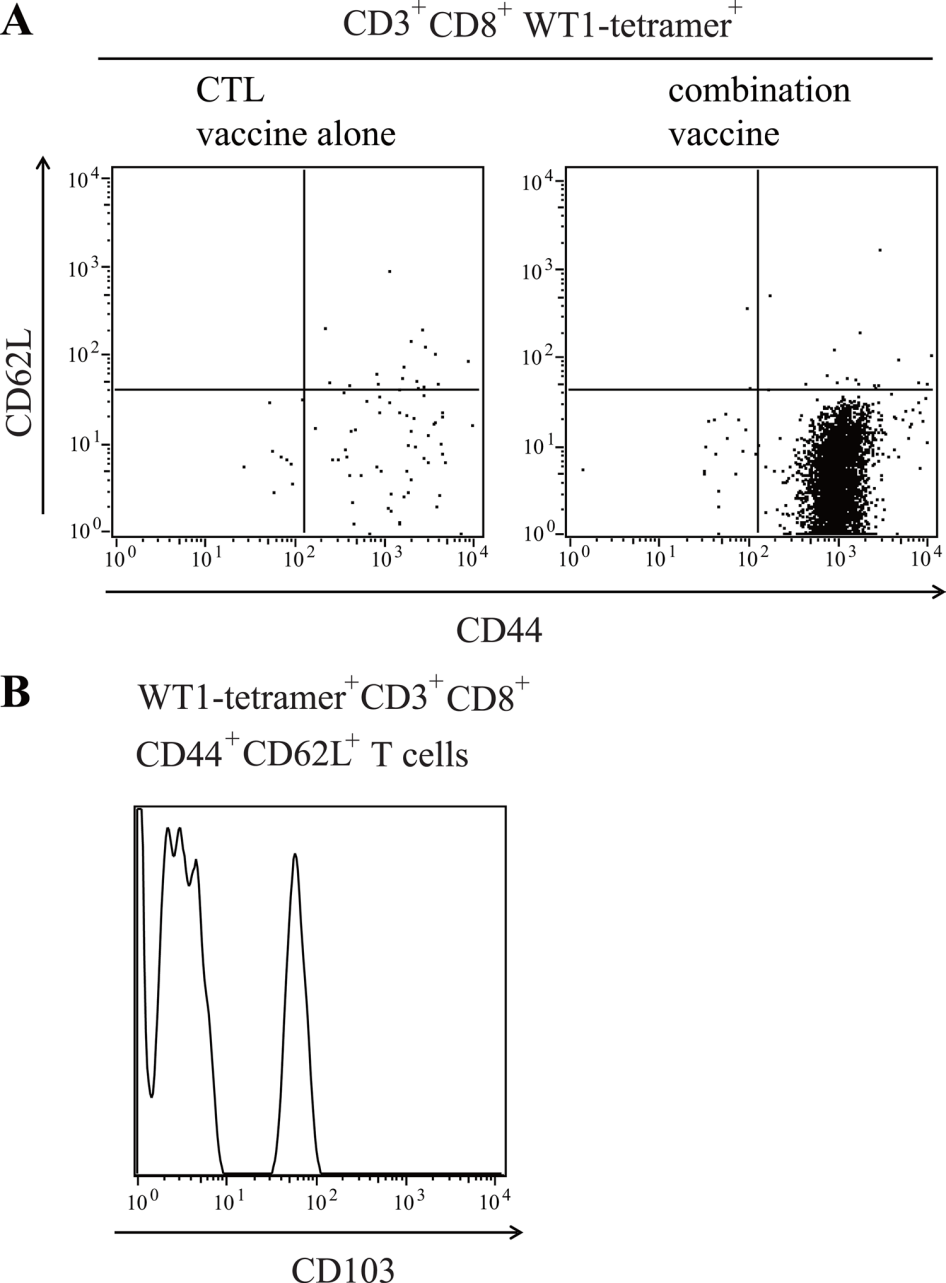
Infiltration of WT1-specific resident memory T cells into the tumors of mice treated with the WT1 combination vaccine (**A**) Representative results of flow cytometry analysis for CD62L and CD44 expression of tumor infiltrating WT1-tetramer^+^ CD3^+^CD8^+^ T cells. (**B**) Representative results of flow cytometry analysis for the expression of CD103 on WT1-tetramer^+^ CD3^+^ CD8^+^CD44^+^CD62^+^ T cells in tumors of mice treated with the combination vaccine.

WT1 tetramer^+^ CD8^+^ T cells infiltrated into the tumors were stained for CD44 and CD62L expression to further characterize them (Figure 4A). CD44^+^ CD62^-^ effector phenotype T cells were much more in the tumors of the mice treated with the WT1 combination vaccine than in those of the mice treated with the WT1 CTL vaccine alone, while the infiltration of CD44^+^ CD62L^+^ memory phenotype T cells was similar. The infiltration of CD44^-^ CD62L^+^ naïve phenotype T cells was very little in both tumors. Furthermore, approximately 40% of WT1 tetramer^+^ CD44^+^ CD62L^+^ effector-memory phenotype CD8^+^ T cells that infiltrated into the tumors of the mice treated with the WT1 combination vaccine expressed CD103, which was a good marker of resident memory T cells, suggesting the *in situ* production of effector T cells from these resident memory T cells in tumors (Figure 4B).

### Tumor infiltrating WT1-specific CD8^+^ T cells showed the clonal expansion

**Figure 5:**
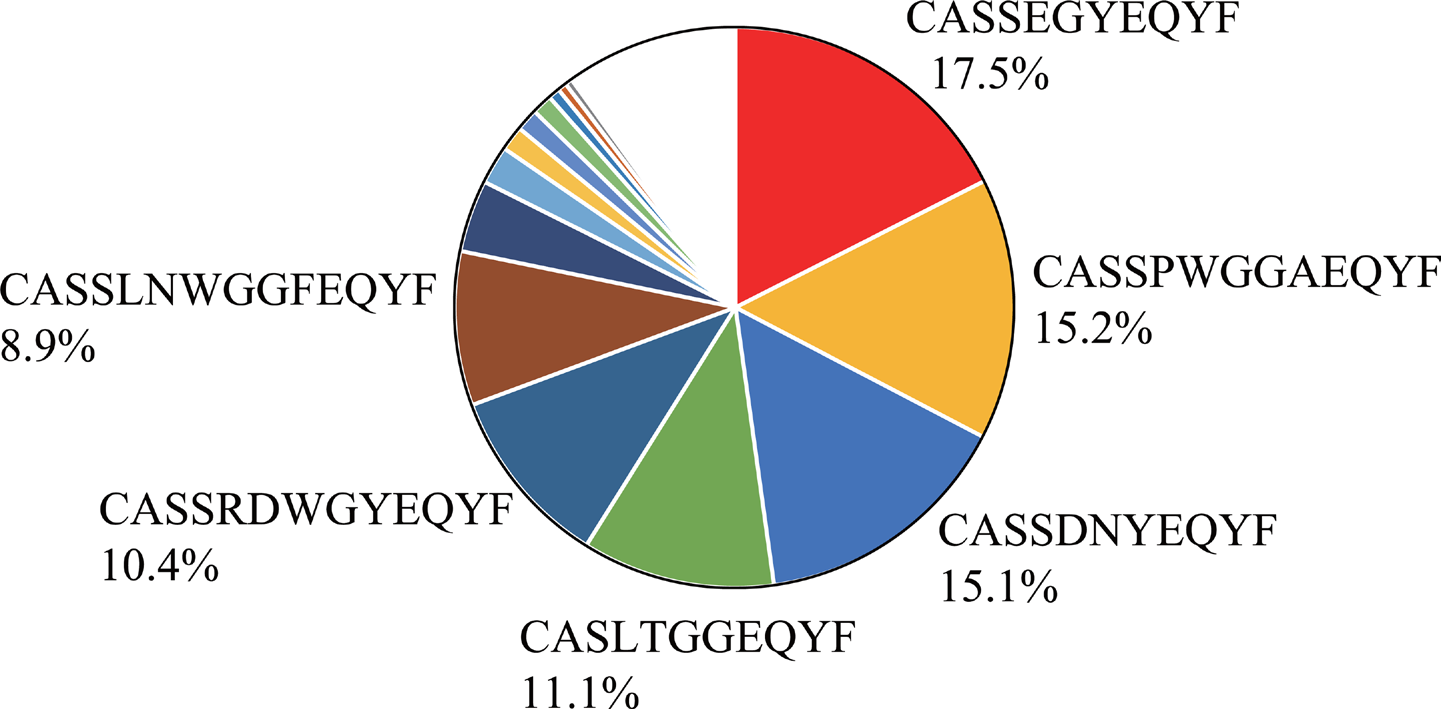
Oligoclonality of tumor infiltrating WT1-tetramer^+^ CD8^+^ T cells T-cell receptor repertoire profiling. Pie chart of the frequencies of WT1-specific CD8^+^ T cell clones is shown. Different colors represent different clones.

To examine whether the WT1-specific CTLs infiltrated into the tumors were clonally expanded, clonality of the CTLs in tumor of the mouse in which the frequencies of WT1-specific CTLs were 26.9% (Figure 3C) was analyzed by sequencing the CDR3 region of TCR-β. It showed their clonal expansion and 3 clones occupied about half of the WT1-specific CTLs (Figure 5).

## DISCUSSION

The present study is the first demonstration using tumor-associated antigen (TAA)-derived CTL and helper peptides in an *in vivo* mouse model that combination vaccine with TAA-derived CTL and helper peptides induced the higher infiltration of the TAA-specific CD8^+^ CTLs than vaccination with the CTL peptide alone in tumor sites. Our data indicated the importance of activation of TAA-specific CD4^+^ helper T cells in tumor immunity.

The frequencies of WT1-specific CD8^+^ T cells in tumor infiltrating CD8^+^ T cells were 0.32% and 8.8% on average in tumors of mice treated with the WT1 CTL peptide vaccine alone or with the combination vaccine of WT1 CTL and helper peptides, respectively, and thus were approximately 27 times higher in the latter than in the former. In a certain mouse treated with the combination vaccine, WT1-specific CD8^+^ T cells accounted for up to as much as 26.9% of tumor infiltrating CD8^+^ T cells (Figure 3C). High frequencies of CD8^+^ T cell response to WT1 peptide that was endogenous and self-antigen was surprising, although such high frequencies of CD8^+^ T cell response to exogenous and non-self-antigens, such as the antigens of bacterium and viruses, were usual.

There are at least two reasonable explanations for the increased infiltration of the effector type WT1-specific CD8^+^ T cells into the tumors, which probably induced microscopic necrotic lesions in them, in mice vaccinated with both WT1 CTL and helper peptides, compared to mice vaccinated with WT1 CTL peptide alone. One explanation is the increased infiltration of the WT1-specific effector CD8^+^ T cells via enhancement by WT1-specific CD4^+^ T cells of their homing into the tumor from outside. These WT1-specific effector CD8^+^ T cells are called “short-lived effector T cells”, which expand in the draining lymph nodes and home into tumors from peripheral blood. The mechanism to enhance the homing of CTLs by helper peptide vaccines was reported to be due to the secretion of IFN-γ from the tumor infiltrating Th1 CD4^+^ T cells [15]. The secreted IFN-γ induces the production of INF-γ-responsive chemokines such as CXCLs 9,10 and 11 [16, 17] from the surrounding cells except T cells, and activated CD8^+^ T cells with CXCR3, which is a receptor for the chemokines, were gravitated the sites with high concentration of the chemokines. The secreted IFN-γ also induces the expression of various types of ligands for T cell homing receptors on tumor-associated endothelium, and as the result, the activated CD8^+^ T cells were gravitated into the tumors, resulting in the formation of microscopic necrotic lesions [18]. Another is that pre-existing, CD44^+^CD62L^+^CD103^+^ WT1-specific T_RM_ cells [19] (see Figure 4B) expanded by the help of WT1-specific CD4^+^ T cells and produced a lot of WT1-specific effector CD8^+^ T cells in tumors. Although how T_RM_ cells worked in tumor immunity and whether T_RM_ cells could truly expand in tumors were not yet clarified, it was reported that a high frequency of T_RM_ cells were correlated with favorable clinical outcome in patients with cancers [20]. It is also known that T_RM_ cells could be expanded in an antigen-specific manner in the peripheral tissues on re-infection of pathogens [21]. It appears that actually, at least two events described above simultaneously occurred and multiple microscopic necrotic lesions were formed. The frequencies of WT1-specific CD8^+^ T cells were 27 times higher in tumors of mice treated with the combination vaccine than in those treated with the CTL vaccine alone, although the frequencies of the whole infiltrated CD8^+^ T cells were similar in both tumors. Therefore, in the former explanation, it is difficult to explain this phenomenon that WT1-specific CD8^+^ T cells very biasedly infiltrated outstripping the infiltrating bystander CD8^+^ T cells in tumor of mice treated with the combination vaccine. Therefore, the latter explanation that WT1-specific T_RM_ cells expanded by the help of WT1-specific CD4^+^ T cells and produced a lot of WT1-specific effector CD8^+^ T cells in tumors is plausible for the biased WT1-specific CD8^+^ T cell infiltration with clonal expansion. In this case, the accumulated CD11c^+^ cells that surrounded the microscopic necrotic lesions played an important role in the *in situ* interaction between the DCs and WT1-specific CD4^+^ and/or CD8^+^ T cells, resulting in the activation of the CD4^+^ and/or CD8^+^ T cells. Therefore, our present results should provide us with a novel concept that the expansion of effector T cells derived from T_RM_ in tumor sites was important for the success in tumor immunotherapies and that the helper peptide vaccine played an essential role in this step through the induction of the antigen-specific helper CD4^+^ T cells in the vicinity of T_RM_. Further studies are needed to completely elucidate how vaccination with antigen-specific helper peptide contributes to accumulation of antigen-specific CTLs in tumor sites, leading to tumor eradication.

## MATERIALS AND METHODS

### Mice

CD45.1 and CD45.2 C57BL/6J mice (from 6- to 8- week old, male) were purchased from CREA Japan (Osaka, Japan), and all animal experiments in this study were approved by the administrative panel on laboratory animal care in Osaka University.

### *In vivo* tumor model and treatment

C1498-mWT1 leukemia cell lines and WT1 peptide vaccines were prepared as previously reported [14]. Shortly, WT1_126–134_ (RMFPNAPYL) and WT1_35–52_ (WAPVLDFAPPGASAYGSL, 18a.a.) peptides were used for WT1 CTL and WT1 helper peptides, respectively. Both peptides were synthesized by SIGMA Genosys (Ishikari, Japan) with the purity of more than 80%. These peptides were emulsified with Montanide ISA 51, an incomplete Freund’s adjuvant (Seppic S.A., Orsay, France) and used for vaccine therapy. C1498-mWT1 cells (1.8 × 10(4) cells/g of mouse weight) were subcutaneously transplanted at the right lower abdomen. WT1 peptide vaccines were prepared as oil-in-water emulsion with Montanide ISA 51 and subcutaneously injected near the left axillary. Tumor size was measured every 2 days, and tumors were resected and analyzed when the tumor size exceed 1 cm.

### Hematoxylin and eosin (HE) staining and immunohistochemistry

Tumor samples were fixed in 4% paraformaldehyde at 4°C overnight and embedded in paraffin. For HE staining, sections were deparaffinized and then stained with hematoxylin for 8 minutes. After washing with flowing water, they were stained with eosin for 2 minutes. For immunohistochemistry, sections were pre-treated with heat mediated antigen retrieval with Dako Target Retrieval Solution (Ph6.0) (DakoCytomation, Copenhagen, Denmark). After the peroxidase-blocking with Dako peroxidase-blocking solution (DAKO), the sections were incubated with anti-mouse CD4 monoclonal antibody (Sino Biological, Beijing, China) or anti-mouse CD8 polyclonal antibody (Bioss Antibodies, Boston, MA, USA) or Anti-ITGAX rabbit polyclonal antibody (OriGene Technologies, Rockville, MD, USA) overnight. Afterwards, Dako Envision ^TM+^ system, HRP (DAKO) was used for secondary antibody and visualization.

### Analysis of tumor infiltrating immune cells

Resected tumors were weighed, mechanically minced and finally taken to cell suspension in RPMI 1640 medium with the enzymes of tumor dissociation kit (Miltenyi Biotec, Gradbach, Germany) mounted on the gentleMACS Octo Dissociator (Miltenyi Biotec). Cell suspension was filtered through a 100 μm cell strainer to remove the debris, and approximately 1 × 10^6^ cells were stained with anti-mouse CD45.1 FITC (A20: eBioscience, San Diego, CA, USA) and anti-mouse CD45.2 PE (104: Biolegend, San Diego, CA, USA) to discriminate tumor infiltrating immune cells from implanted tumor cells. CD45.1 positive cells were selected from the rest of the cell suspensions by using MagniSort Mouse CD45.1 Positive Selection Kit (Thermo Fisher Scientific, Waltham, MA, USA). Then, approximately 1 × 10^6^ cells were stained with Brilliant Violet 510 anti-mouse CD45.2 antibody (104: Biolegend), FITC anti-mouse CD3 antibody (17A2: Biolegend), PE/Cy7 anti-mouse CD4 antibody (RM4-5: Biolegend), Anti-mouse CD8 mAb-Alexa Fluor 647 (KT15: MBL, Aiti, Japan), APC/Cy7 anti-mouse NK-1.1 antibody (PK136: Biolegend) and H-2Db WT1 126-134 tetramer-RMFPNAPYL-PE (MBL) to analyze the frequencies of NK cells, CD4 and CD8 T cells among tumor infiltrating immune cells. Approximately 1 × 10^6^ cells of the rest were stained with Brilliant Violet 510 anti-mouse CD45.2 antibody, FITC anti-mouse/human CD11b antibody (M1/70: Biolegend), APC anti-mouse CD11c antibody (N418: Biolegend), APC/Cy7 anti-mouse Ly-6G antibody (1A8: Biolegend) and Brilliant Violet 421 anti-mouse Ly-6C antibody (HK1.4: Biolegend) to analyze the frequencies of DC, PMN-MDSC and M-MDSC cell among tumor infiltrating immune cells. For analysis of tumor infiltrating WT1-specific CTLs, CD8^+^ cells were selectively collected from the cell suspensions by using MagniSort Mouse CD8 Positive Selection Kit (Thermo Fisher Scientific), and stained with Brilliant Violet 510 anti-mouse CD45.2 antibody, Pacific Blue anti-mouse CD103 (2E7, Biolegend), FITC anti-mouse CD3 antibody, PE/Cy7 anti-mouse/human CD44 antibody (IM7, Biolegend), Anti-mouse CD8 mAb-Alexa Fluor 647, APC/Cy7 anti-mouse CD62L antibody (MEL-14, Biolegend) and H-2Db WT1 126-134 tetramer-RMFPNAPYL-PE.

### Intracellular cytokine staining of tumor infiltrating CD4^+^ T cells

CD4^+^ cells were selectively collected from tumor cell suspension by using MagniSort Mouse CD4 Positive Selection Kit (Thermo Fisher Scientific), stimulated with WT1_35–52_ peptide, and then cultured in complete medium with 20 IU/ml interleukin-2 (Shionogi Biomedical Laboratories, Osaka, Japan). Thirteen days later, the cultured tumor infiltrating CD4^+^ T cells were co-cultured with antigen presenting cells, which were CD45.2 C57BL/6J mouse splenocytes pulsed with WT1_35–52_ peptide and irradiated for 30 minutes, in complete medium containing with 10 μg/ml Brefeldin A (SIGMA-Aldrich, St. Louis, MO, USA) for 4 hours. After 4 hours of culture, the cells were stained with FITC anti-mouse CD3 antibody, APC-eFluor 780 anti-mouse CD4 antibody (RM4-5: eBioscience), PE anti-mouse CD8α antibody (53-6.7: eBioscience), Pacific Blue anti-mouse CD45.1 antibody (A20: Biolegend) and Brilliant Violet 510 anti-mouse CD45.2 antibody. Then, the cells were fixed, permeabilized by Cytofix/Cytoperm solution (BD Bioscience, San Jose, CA, USA), and stained with PE/Cy7 anti-mouse IFN-γ (XMG1.2: Biolegend) and APC anti-mouse TNF-α (MP6-XT22: Biolegend).

### T-cell receptor repertoire analysis of tumor infiltrating WT1-tetramer^+^ CD8^+^ T cells

WT1-tetramer^+^ CD3^+^CD8^+^ T cells were FACS-sorted from tumor cell suspension. The libraries were prepared by using SMARTer Mouse TCR a/b Profiling Kit (Takara Bio Inc, Siga, Japan), and sequenced on the illumine MiSeq system. Results were analyzed by MIGMAP software.

### Statistical analysis

Two-sample *t*-test was used for the statistical analysis in all experiments. Differences were defined as statistically significant when *p* values were less than 0.05.
